# Next-generation sequencing of the human *TRPV1* gene and the regulating co-players *LTB4R* and *LTB4R2* based on a custom AmpliSeq^™^ panel

**DOI:** 10.1371/journal.pone.0180116

**Published:** 2017-06-28

**Authors:** Dario Kringel, Marco Sisignano, Sebastian Zinn, Jörn Lötsch

**Affiliations:** 1 Institute of Clinical Pharmacology, Goethe - University, Frankfurt am Main, Germany; 2 Fraunhofer Institute of Molecular Biology and Applied Ecology - Project Group Translational Medicine and Pharmacology (IME-TMP), Frankfurt am Main, Germany; Duke University School of Medicine, UNITED STATES

## Abstract

**Background:**

Transient receptor potential cation channel subfamily V member 1 (TRPV1) are sensitive to heat, capsaicin, pungent chemicals and other noxious stimuli. They play important roles in the pain pathway where in concert with proinflammatory factors such as leukotrienes they mediate sensitization and hyperalgesia. TRPV1 is the target of several novel analgesics drugs under development and therefore, *TRPV1* genetic variants might represent promising candidates for pharmacogenetic modulators of drug effects.

**Methods:**

A next-generation sequencing (NGS) panel was created for the human *TRPV1* gene and in addition, for the leukotriene receptors BLT1 and BLT2 recently described to modulate TRPV1 mediated sensitisation processes rendering the coding genes *LTB4R* and *LTB4R2* important co-players in pharmacogenetic approaches involving *TRPV1*. The NGS workflow was based on a custom AmpliSeq^™^ panel and designed for sequencing of human genes on an Ion PGM^™^ Sequencer. A cohort of 80 healthy subjects of Western European descent was screened to evaluate and validate the detection of exomic sequences of the coding genes with 25 base pair exon padding.

**Results:**

The amplicons covered approximately 97% of the target sequence. A median of 2.81 x 10^6^ reads per run was obtained. This identified approximately 140 chromosome loci where nucleotides deviated from the reference sequence GRCh37 hg19 comprising the three genes *TRPV1*, *LTB4R* and *LTB4R2*. Correspondence between NGS and Sanger derived nucleotide sequences was 100%.

**Conclusions:**

Results suggested that the NGS approach based on AmpliSeq^™^ libraries and Ion Personal Genome Machine (PGM) sequencing is a highly efficient mutation detection method. It is suitable for large-scale sequencing of *TRPV1* and functionally related genes. The method adds a large amount of genetic information as a basis for complete analysis of TRPV1 ion channel genetics and its functional consequences.

## Introduction

The transient receptor potential (TRP) family comprises several non-selective cation channels [1] enabling or inhibiting the transmembrane transport of several ions. Various members of this ion channel family are expressed at nociceptors and via their excitation by chemical, thermal or mechanical stimuli involved in the perception of pain [2]. This makes them primary candidates for the discovery of novel analgesic drugs [3]. A query of the Thomson Reuters “Drugs and Biologics Search Tool” (http://integrity.thomsonpharma.com) in June 2016 indicated that by far the most frequently regarded TRP member in analgesic drug development is TRP cation channel, subfamily V, member 1 (TRPV1 [4]) for which more than 200 agonists or antagonists are currently under development, which bases on the concept that endogenous agonists or sensitizers acting on TRPV1 provide a major contribution to pathophysiological pain conditions [5, 6]. The pharmacological modulation of this mechanism employs (i) the approach of direct antagonism of the TRPV1 ion channel, (ii) the exposure to agonists such as capsaicin that initially activates TRPV1 but upon prolonged exposure induces a deactivation via a calcineurin-dependent channel dephosphorylation and desensitization [7] and (iii) to prevent a sensitization and hyperactivation of the TRPV1 channel [8].

Given the importance of TRPV1 in pain and analgesic drug discovery and development, *TRPV1* genetics move into a focus of pharmacogenetic interest. A modulation of the effects of TRPV1 targeting analgesics is supported by observations that intronic *TRPV1* variants were associated with insensitivity to capsaicin [9] while the coding *TRPV1* variant rs8065080 was associated with altered responses to experimentally induced pain [10]. Moreover, gain-of-function mutations in TRPV1 have been associated with increased pain sensitivity [11], for which TRPV1 antagonists would enable a specific pharmacogenetics-based personalized cure. Hence, genetic variation of human *TRPV1* is in a focus of pain and analgesic research. With the broader availability of next generation sequencing (NGS) [12], a limitation to already investigated variants has fallen in favor of unrestricted access to the whole genetic information in agreement with the wider acceptance of whole genomic information as a valuable method in clinical research [13].

In this report, the evaluation of a new NGS method based on a custom AmpliSeq^™^ library and Ion Torrent sequencing for the fast detection of genetic variations in the human *TRPV1* gene is described. However, preclinical evidence indicates that leukotriene B4 mediates the inflammation via TRPV1 [14] and that the nociceptive function of TRPV1 is modulated by the activation of leukotriene receptors BLT1 and BLT2 [8] that are highly expressed in TRPV1 expressing dorsal root ganglion neurons. Both receptors form an antagonistic sensitizing system and have opposing roles in TRPV1 sensitisation. This renders them important co-players in pharmacogenetic approaches at analgesics aiming at modulation of the function of TRPV1. To provide a comprehensive basis for pharmacogenetic assessments of TRPV1 modulators, the present NGS panel was extended with human *LTB4R* and *LTB4R2* genes that code for the leukotriene receptors of present interest.

## Methods

### DNA template preparation and amplification

The investigation followed the Declaration of Helsinki on Biomedical Research Involving Human Subjects and was approved by the Ethics Committee of the Medical Faculty of the Goethe-University, Frankfurt, Germany. All participating subjects had provided informed written consent. Genomic DNA was available from venous blood samples drawn from a random sample of 80 healthy volunteers of Western European descent according to self-assignment. DNA was extracted from 200 μl blood on a BioRobot EZ1 workstation applying the blood and body fluid spin protocol provided in the EZ1 DNA Blood 200 μl Kit (Qiagen, Hilden, Germany).

Exomic genotyping was performed for the *TRPV1* gene (NCBI ID 7442), located on chromosome 17 and encoding for the TRPV1 ion channel and for the *LTB4R* and *LTB4R2* genes (NCBI IDs 1241 and 56413), both located on chromosomes 14 and encoding for leukotriene B4 receptors BLT1 and BLT2. A multiplex PCR amplification strategy for the coding genes sequences was accomplished online (Ion Ampliseq^™^ Designer; http://www.ampliseq.com) to amplify the target region specified above (for primer sequences, see S1 Table) with 25 base pair exon padding. After comparison of several primer design options, the design providing the maximum target sequence coverage was chosen. The ordered amplicons covered 97.02% of the target sequence. A total of 10 ng DNA per sample were used for the target enrichment by a multiplex PCR and each DNA pool was amplified with the Ion Ampliseq^™^ Library Kit in conjunction with the Ion Ampliseq^™^ “custom Primer Pool”—protocols according to the manufacturer procedures (Life Technologies, Darmstadt, Germany).

After each pool had undergone 17 PCR cycles, the PCR primers were removed with FuPa Reagent and the amplicons were ligated to the sequencing adapters with short stretches of index sequences (barcodes) that enabled sample multiplexing for subsequent steps (Ion Xpress^™^ Barcode Adapters Kit; Life Technologies). After purification with AMPure XP beads (Beckman Coulter, Krefeld, Germany), the barcoded libraries were quantified with a Qubit^®^ 2.0 Fluorimeter (Life Technologies, Darmstadt, Germany) and normalized for DNA concentration to a final concentration of 20 pmol/L using the Ion Library Equalizer^™^ Kit (Life Technologies, Darmstadt, Germany). Equalized barcoded libraries from 11 to 40 samples at a time were pooled. To clonally amplify the library DNA onto the Ion Sphere Particles (ISPs; Life Technologies, Darmstadt, Germany), the library pool was subjected to emulsion PCR by using an IT OneTouch template kit on an IT OneTouch system (Life Technologies, Darmstadt, Germany) following the manufacturer’s protocol.

### Sequencing

Enriched ISPs which carried many copies of the same DNA fragment were subjected to sequencing on an Ion 318 Chip to sequence pooled libraries with eleven to twelve samples. The 318 chip was chosen (instead of the low-capacity 314 or the middle-capacity 316 chip) to obtain a high sequencing depth of coverage which was averagely of 50x which means that, each base has been sequenced 50 times, when 40 samples were loaded. Sequencing was performed using the sequencing kit (Ion PGM 200 Sequencing Kit; Life Technologies, Darmstadt, Germany) as per the manufacturer’s instructions with the 200-bp single-end run configuration.

### Bioinformatics generation of sequence information

The raw data (unmapped BAM-files) from the sequencing runs were processed using Torrent Suite Software (Version 4.4.2, Life Technologies, Darmstadt, Germany) to generate read alignments which are filtered by the software into mapped BAM-files using the reference genomic sequence (hg19) of target genes. Variant calling was performed with the Torrent Variant Caller Plugin using as key parameters: minimum quality = 10, minimum coverage = 20, and minimum coverage on either strand = 3. The annotation of called variants was done using the Ion Reporter Software (Version 5.0; Life Technologies, Darmstadt, Germany) and the variant classification tool of the SNP and Variation Suite software (Version 8.4.4; Golden Helix, Bozeman, MT, USA) for the VCF (variant call format) files that contained the nucleotide reads and the GenomeBrowse^®^ software (Version 2.0.4, Golden Helix, Bozeman, MT, USA) to map the sequences to the reference sequences GRCh37 g1k (dated February 2009).

On the basis of the observed allelic frequency, the expected number of homozygous and heterozygous carriers of the respective SNP (single nucleotide polymorphism) was calculated using the Hardy-Weinberg equation. Indicating that the study sample corresponded to a random sample of subjects, Fisher’s exact test [15] was used as proposed previously [16]. Only variants within the Hardy-Weinberg equilibrium were retained. The SNP and Variation Suite software (Version 8.4.4; Golden Helix, Bozeman, MT, USA) was used for the analysis of sequence quality, coverage and for variant identification.

### Method validation

Method validation was accomplished by means of Sanger sequencing [17, 18] in an independent external laboratory (Eurofins Genomics, Ebersberg, Germany). For the detected variant type, i.e., single nucleotide polymorphisms (SNV), nucleotide insertions (Ins) and nucleotide deletions (Del), the variant with the highest frequency of the rare allele was chosen for external sequencing: 17:3493769-SNV, 17:3496181_Ins, 17:3512619_Del. In addition, the variant 17:3480447-SNV, which is the functional rs8065080 SNP previously associated with altered pain sensitivity [10], was added accommodating the present context of analgesics’ pharmacogenetic. Amplification of the respective DNA segments was done using PCR primer pairs (forward, reverse) of (i) 5´-CCATGTTGCGTCTCTCGATG-3´ and 5´-CAACCCGTTATTTCCTGTTCCCA-3´ (ii) 5´- CTCAGAGGTGAGCAGGCCTAGC -3´ and 5´- AAGGCCAGGATGCTTGACAGATG -3´, (iii) 5´- AAGGCACAAGACTCTGGAAGAAT-3´ and 5´- CGAGTTTGGGAAGCAGTCGTAT-3´ and (iv) 5´- ACCCAGTGCCTTCTCATTCAG-3´ and 5´- CACGTTCTCAAGACGCATCC-3´. Results of Sanger sequencing were aligned with the genomic sequence and analyzed using Chromas Lite^®^ (Version 2.1.1, Technelysium Pty Ltd, South Brisbane, Australia) and the GenomeBrowse^®^ (Version 2.0.4, Golden Helix, Bozeman, MT, USA) was used to compare the sequences obtained with NGS or Sanger techniques.

## Results

**Fig 1:**
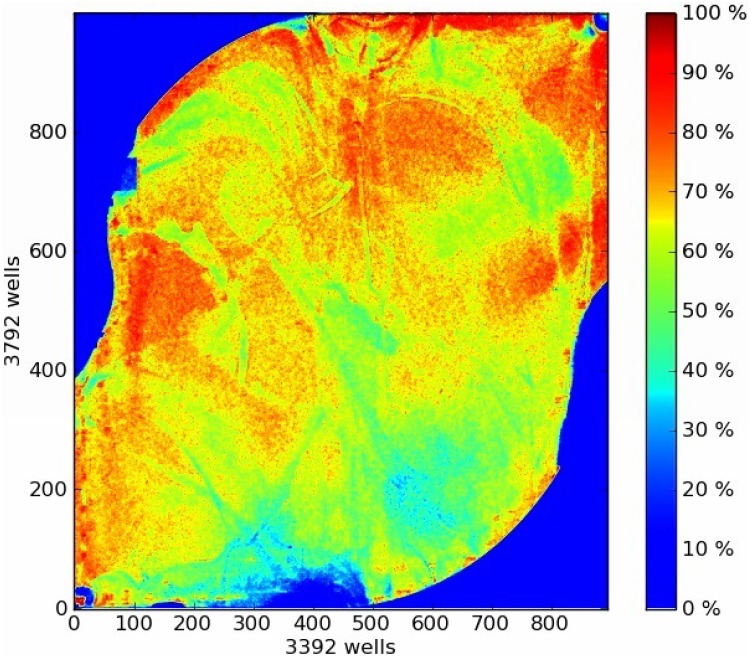
Pseudo-color image of the Ion 318^™^ v2 chip plate showing percent loading across the physical surface. This sequencing run had a 70% loading, which ensures a high ISP density. Every 318 chip contains more than 6 million wells and the color scale on the right side conduces as a loading indicator. Deep red coloration stays for a 100% loading, which means that every well in this area contains an ISP (templated and non-templated) whereas deep blue coloration implies that the wells in this area are empty.

**Fig 2:**
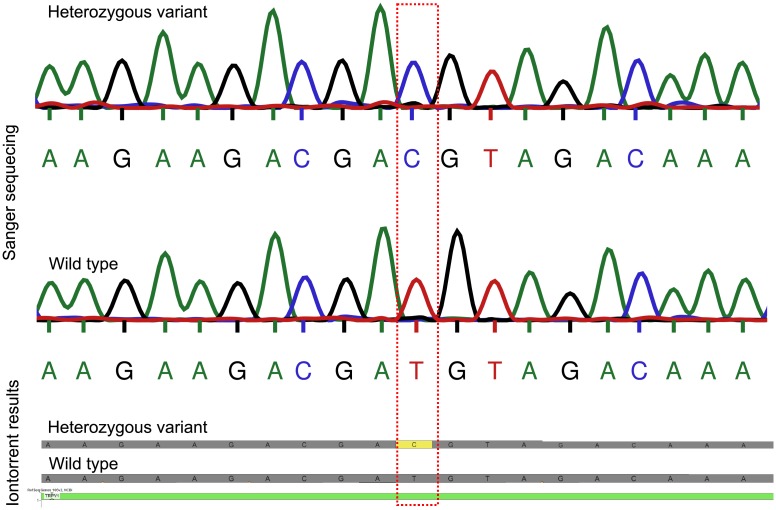
Alignment of the ion torrent sequence of the *TRPV1* gene illustrated by Golden Helix Genome Browse^®^ readout versus the same sequence according to a Sanger electrophereogram. Highlighted is the coding *TRPV1* variant rs8065080 as a heterozygous mutation and a non-mutated wild type.

The NGS assay of human *TRPV1*, *LTB4R2* and *LTB4R* genes was established on 80 genomic DNA samples obtained from a random selection of healthy subjects of Caucasian ethnicity. As proposed previously [19], only exons and their boundary sequences for which read-depths > 20 for each nucleotide could be obtained were considered as successfully analyzed. Applying this criterion, complete or nearly complete coverage of the relevant sequences was obtained (Table 1; for details on missing variants, see S2 Table). The sequencing of the whole cohort required two separate runs with each 40 patients’ samples. Coverage statistics (Table 1) were comparable between both runs and were in the range of accepted quality criteria [20–22]. During the runs, a median of 2.81 · 10^6^ reads per run was generated. The mean depth was near from 200 reads, the mean read length evaluated 198 bases and average chip loading was 66% (Fig 1). To ensure a high density of ISPs on a chip and hence, a high sequencing output, the chip loading value should be ≥ 60%. The observed NGS results agreed with the results obtained with conventional sequencing of random samples (Fig 2). In all validation samples, the correspondence between NGS and Sanger derived nucleotide sequences was 100%, all of the tested nucleotide variants could be verified.

**Table 1:** AmpliSeq^™^ amplicons and coverage details of the human *LTB4R2*, *LTB4R and TRPV1* NGS assay.

Gene	Chr*	Chr start	Chr end	Amplicons	Total bases	Covered bases	Coverage	Sum (total, covered, %)
*LTBR42*	Chr14	34634693	34635880	8	1187	1187	1.000	3520, 3427, 98.3%
		34636968	34637111	1	143	143	1.000	
		34636968	34637134	1	166	166	1.000	
		34637238	34637442	2	204	204	1.000	
		34637191	34637442	2	251	251	1.000	
		34637578	34637848	2	270	251	0.930	
		34637518	34637848	2	330	256	0.776	
		34634693	34635880	8	1187	1187	1.000	
*LTBR4*	Chr14	34637238	34637442	2	204	204	1.000	5683, 5117, 97%
		34637191	34637442	2	251	251	1.000	
		34637578	34637848	2	270	251	0.930	
		34637518	34637848	2	330	256	0.776	
		34634693	34635880	8	1187	1187	1.000	
		34637238	34637442	2	204	204	1.000	
*TRPV1*	Chr17	24636968	24637111	1	143	143	1.000	23787, 21859, 98.8%
		24636968	24637134	1	166	166	1.000	
		24637238	24637442	2	204	204	1.000	
		24637191	24637442	2	251	251	1.000	
		24636968	24637134	1	166	166	1.000	
		24637238	24637442	2	204	204	1.000	
		24637191	24637442	2	251	251	1.000	
		24636968	24637134	1	166	166	1.000	
		24637238	24637442	2	204	204	1.000	
		24637191	24637442	2	251	251	1.000	
		24636968	24637134	1	166	166	1.000	
		24637238	24637442	2	204	204	1.000	
		24637191	24637442	2	251	251	1.000	
		24637191	24637442	2	251	251	1.000	
		24636968	24637134	2	270	251	0.930	
		24637238	24637442	2	330	256	0.776	
		24637191	24637442	8	1187	1187	1.000	
		24636968	24637134	2	251	251	1.000	
		24637238	24637442	2	270	251	0.930	
		24637191	24637442	2	330	256	0.776	
		24636968	24637134	8	1187	1187	1.000	

*: Chr: Chromosome.

**Fig 3:**
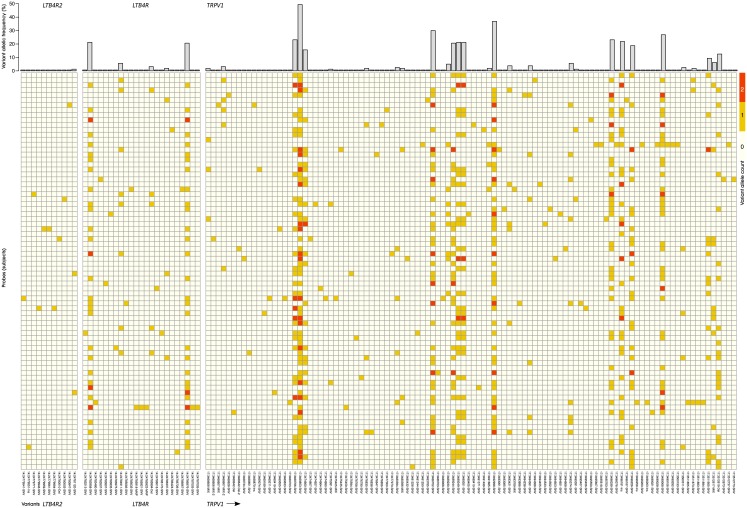
*LTB4R2*, *LTB4R* and *TRPV1* genetic pattern of 80 healthy volunteers of Caucasian ethnicity. The mosaic plot shows the occurrence of variants (lines) per DNA sample (columns) as vectors of a length corresponding to the number of gene loci in which a non-reference nucleotide was found in any sample of the whole cohort. The vectors are composed of information about the number of non-reference alleles found at the respective locus in the respective sample, color codes as white, 0 non-reference alleles = wild type genotype, yellow, heterozygous, and red, 2 non-reference alleles). The bar plot on the top shows the number of variant alleles found in the cohort.

Following elimination of nucleotides agreeing with the standard human genome sequence GRCh37 g1k (dated February 2009), the result of the NGS consisted of a vector of nucleotide information about the *LTB4R2*, *LTB4R* and *TRPV1* genes for each individual DNA sample (Fig 3). This vector had a length equaling the set union of the number of chromosomal positions in which a non-reference nucleotide had been found in any probe of the actual cohort of randomly chosen healthy subjects. Specifically, a total of 156 genetic variants was found, of which 11, 28 and 117 were located in the *LTB4R2*, *LTB4R* and *TRPV1* genes, respectively (Fig 3). Of the observed variants, 38 were located in coding parts of the genes (Table 2), 56 were located in introns, 33 in the 3’-UTR, 16 in the 5’-UTR, 5 variants were assigned to both UTR’s and 8 were located downstream. The nucleotidic and, if present, the resulting amino acid exchanges, of the coding variants are listed in Table 2. The allelic frequencies corresponded to those expected based on the Hardy-Weinberg equilibrium (Fisher’s exact tests: p always > 0.05) and, for variants with reported clinical functional association, the observed allelic frequency was comparable to reported frequencies (Table 3). Most of the observed variants were single nucleotide polymorphisms (n = 135; 9, 25 and 101 in the *LTB4R2*, *LTB4R* and *TRPV1* genes, respectively) whereas classified as mixed polymorphisms (n = 8), nucleotide insertions (n = 6), nucleotide deletions (n = 5) or multinucleotide polymorphisms (n = 2) were more rarely found in the present cohort.

**Table 2:** A list of variants found in the coding parts of the *LTB4R2*, *LTB4R and TRPV1* genes in a random sample of 80 healthy volunteers of Caucasian ethnicity.

Gene	Variant	Chr*	Position	Classification	Exon	Coding	Protein
*LTB4R2*	14:24779946-SNV	14	24779946	Nonsyn SNV	2	c.76T>C	p.Phe26Leu
	14:24779959-SNV	14	24779959	Nonsyn SNV	2	c.89C>T	p.Ala30Val
	14:24779961-SNV	14	24779961	Nonsyn SNV	2	c.91G>A	p.Ala31Thr
	14:24779994-SNV	14	24779994	Nonsyn SNV	2	c.124G>A	p.Val42Met
	14:24780010-Del	14	24780010	Frameshift Del	2	c.140_164del	p.Ala51fs
	14:24780503-SNV	14	24780503	Synonymous	2	c.633C>T	p.=
	14:24780847-SNV	14	24780847	Nonsyn SNV	2	c.977A>G	p.Glu326Gly
*LTB4R*	14:24784911-SNV	14	24784911	Synonymous	2	c.54T>C	p.=
	14:24785083-SNV	14	24785083	Nonsyn SNV	2	c.226C>T	p.His76Tyr
	14:24785633-SNV	14	24785633	Nonsyn SNV	2	c.776T>C	p.Val259Ala
	14:24785784-SNV	14	24785784	Synonymous	2	c.927C>T	p.=
*TRPV1*	17:3474927-SNV	17	3474927	Synonymous	14	c.2238C>T	p.=
	17:3475435-SNV	17	3475435	Nonsyn SNV	13	c.2212G>T	p.Asp738Tyr
	17:3475459-SNV	17	3475459	Nonsyn SNV	13	c.2188G>A	p.Gly730Arg
	17:3475490-SNV	17	3475490	Synonymous	13	c.2157G>A	p.=
	17:3476990-SNV	17	3476990	Synonymous	12	c.2040C>T	p.=
	17:3477000-SNV	17	3477000	Nonsyn SNV	12	c.2030A>G	p.Asn677Ser
	17:3480432-SNV	17	3480432	Nonsyn SNV	11	c.1768G>A	p.Gly590Arg
	17:3480447-SNV	17	3480447	Nonsyn SNV	11	c.1753A>G	p.Ile585Val
	17:3480910-SNV	17	3480910	Synonymous	10	c.1695T>C	p.=
	17:3483785-SNV	17	3483785	Nonsyn SNV	9	c.1513A>G	p.Thr505Ala
	17:3486702-SNV	17	3486702	Nonsyn SNV	8	c.1406C>T	p.Thr469Ile
	17:3486703-SNV	17	3486703	Nonsyn SNV	8	c.1405A>T	p.Thr469Ser
	17:3489068-SNV	17	3489068	Synonymous	7	c.1377T>C	p.=
	17:3491499-SNV	17	3491499	Nonsyn SNV	6	c.1207A>G	p.Ser403Gly
	17:3493200-SNV	17	3493200	Nonsyn SNV	5	c.945G>C	p.Met315Ile
	17:3494361-SNV	17	3494361	Synonymous	3	c.501C>T	p.=
	17:3494388-SNV	17	3494388	Synonymous	3	c.474T>C	p.=
	17:3494533-SNV	17	3494533	Synonymous	2	c.399G>A	p.=
	17:3494562-SNV	17	3494562	Stopgain	2	c.370C>T	p.Gln124*
	17:3494603-SNV	17	3494603	Nonsyn SNV	2	c.329T>C	p.Leu110Pro
	17:3495374-SNV	17	3495374	Nonsyn SNV	1	c.271C>T	p.Pro91Ser
	17:3495391-SNV	17	3495391	Nonsyn SNV	1	c.254A>G	p.Gln85Arg
	17:3495407-SNV	17	3495407	Nonsyn SNV	1	c.238C>T	p.Pro80Ser
	17:3495456-SNV	17	3495456	Synonymous	1	c.189C>T	p.=
	17:3495550-SNV	17	3495550	Nonsyn SNV	1	c.95G>T	p.Arg32Met
	17:3495607-SNV	17	3495607	Nonsyn SNV	1	c.38C>T	p.Ala13Val
	17:3495618-SNV	17	3495618	Nonsyn SNV	1	c.27G>C	p.Leu9Phe

*: Chr: Chromosome.

**Table 3:** A list of human variants of the *LTB4R and TRPV1* genes, found in the present random sample of 80 healthy volunteers of Caucasian ethnicity, for which functional associations in clinical or human experimental settings have been reported.

Gene	Variant	dbSNP^#^ accession number	Allelic frequency [%] (CI*) Present cohort* HAPMAP CEU		Known clinical association	Reference
*LTB4R*	14:24786060-SNV	rs1046587	46.2 (38.7–54)	47.4	Asthma susceptibility	[28]
	14:24786293-SNV	rs4981503	28.1 (21.7–35.4)	-	Asthma susceptibility	[29]
*TRPV1*	17:3469853-SNV	rs4790522	49.4 (41.7–57)	56.2	Bronchial asthma susceptibility	[30, 31, 66]
					Susceptibility to cough	[67]
					Altered pain sensitivity	[35]
	17:3480447-SNV	rs8065080	33.7 (26.9–41.4)	35.8	Altered cold pain sensitivity	[10]
					Painful knee osteoarthritis	[36]
					Altered salt taste perception	[68]
					Higher risk of type 2 diabetes	[33]
	17:3486702-SNV	rs224534	30 (23.4–37.5)	33.5	Sickle cell pain	[69]
	17:3493200-SNV	rs222747	25.6 (19.5–32.9)	18.3	Functional dyspepsia	[34]
					Ménière's disease	[70]
	17:3495391-SNV	rs55916885	1.2 (0.3–4.4)	-	Cerebellar hypoplasia	[71]

*: CI denotes 95% binomial confidence intervals of the allelic frequencies are given in parentheses after the observed frequency.

^#^: Database of Single Nucleotide Polymorphisms (dbSNP). Bethesda (MD): National Center for Biotechnology Information, National Library of Medicine. Available from: http://www.ncbi.nlm.nih.gov/SNP/ [72]

## Discussion

An NGS assay for the exons and regulatory parts of the human genes coding for the TRPV1 ion channel and those coding for its recently associated co-players comprising the leukotriene receptors BLT1 and BLT2 (*LTB4R*, *LTB4R2)*. The NGS assay produced valid nucleotide sequences corresponding to those obtained with the classical Sanger sequencing technique. The NGS assay is suitable for small to large-scale experimental setups aiming at accessing the information about any nucleotide in a study cohort, with a selection of those that differ from the reference nucleotide.

TRPV1 ion channels mediate pain induced by noxious heat (> 43°C) [23]. A most striking phenotype of *Trpv1* –/– mice is a severe deficit of inflammation-induced thermal hyperalgesia [24]. In addition to heat, TRPV1 expression is largely associated with small diameter primary afferent nerve fibers, which are sensitive to various chemical excitants including protons (low pH), capsaicin, lipoxygenase, resiniferatoxin, ethanol, N-arachidonoyl-dopamine and the endogenous cannabinoid anandamide [3, 25]. Based on evidence that TRPV1 channels are necessary for the development of inflammatory hyperalgesia to thermal stimuli [24] their role in pain has been acknowledged for more than two decades [24, 26]. Currently, they are used as target of capsaicin containing analgesics. However, TRPV1 remains a primary candidate for the discovery of novel analgesic drugs [3] and approximately 200 modulators of this target are currently under development (http://integrity.thomsonpharma.com). This establishes a strong future pharmacogenetic context of *TRPV1* considering the increasing acknowledgment that the treatment of pain will benefit from individualized approaches including those based on the patient’s genotype [8].

Research on the genetic variation of variants in human *TRPV1* or leukotriene receptor genes is an active topic that has already provided several clinically relevant functional associations. A query of the 156 genetic variants in various publicly available data sources (Online Mendelian Inheritance in Man” (OMIM^®^) database at http://www.ncbi.nlm.nih.gov/omim, NCBI gene index database at http://www.ncbi.nlm.nih.gov/gene; GeneCards at http://www.genecards.org [27] and the “1000 Genomes Browser” at https://www.ncbi.nlm.nih.gov/variation/tools/1000genomes; all accessed in May 2017) yielded 13 clinical associations (Table 3). The clinical associations included a variety of pathologies such as pain, asthma or osteoarthritis. Specifically, variants in both, *TRPV1* and *LTB4R* have been associated with a higher susceptibility to bronchial asthma [28–32]. Moreover, *TRPV1* variants have been associated with a higher risk of type 2 diabetes [33] or of functional dyspepsia [34]. Finally, of potential importance for a pharmacogenetic modulation of the effects of future analgesics, *TRPV1* variants have been associated with altered pain phenotypes in clinical or human experimental settings [10, 35, 36]. This fits to the particular role of TRPV1 as a major target for novel analgesic drugs under development.

Winter and colleagues recently created an overview of site-directed mutagenesis studies on *Trpv1* receptor in rodents [37]. Their study summarized information about several mutated sites along the *Trpv1*, which influenced the effect or binding of different compounds like agonists, antagonists, and channel blockers and alter the responsiveness to heat and influence the regulation of the receptor function. Of peculiar interest is the c-terminus part of the receptor, because it contained several mutations implicated in binding of capsaicin. To reference this information to our study, we took out an alignment blast with http://www.uniprot.org/blast/, which is a search tool to find regions of local similarity between sequences and can be used to infer functional and evolutionary relationships between sequences revealed that *TRPV1* is highly conserved. With the present NGS assay, several functional SNPs could be identified in the coding area of *TRPV1*; one variant (17:3477000-SNV) was located in exactly the c-terminus area mentioned above. On this basis, the impact of this variant on nociception can be prospectively studied.

A pharmacogenetic modulation of the effects of TRPV1-targeting analgesics is supported by evidence of associations of rare and of common variants in the human *TRPV1* gene with pain-related clinical phenotypes. Based upon the direction of change of each phenotype and cumulative changes in each SNP, three functional categories of *TRPV1* variants were proposed: gain of function (hTRPV1 Q85R, P91S, and T469I), loss of function (I585V), and mixed (M315I) [38]. These *in vitro* results support clinical observations of *TRPV1* genotypic effects. A Korean subject who was insensitive to capsaicin and displayed mRNA and protein expression levels of TRPV1 reduced by 50% from average subjects was found to carry seven intronic *TRPV1* single nucleotide polymorphisms (SNPs) [9]. Similarly, women carrying a coding *TRPV1* variant were found to be less sensitive to cold [10]. The association possibly involves interactions among TRP channels [39] based on evidence that TRPA1 channels are often co-expressed with heat (> 43°C [4]) gated TRPV1 [40, 41]) and the channels act in concert. TRPV1 can oligomerize with other TRP family subunits including TRPV3 and TRPA1 [42–44] and the heteromerization can affect the calcium signaling pathways of TRPA1 homomers [44]. While heat hyperalgesia was initially attributed solely to TRPV1, currently TRPA1 and TRPV1 are regarded to be regulated downstream of PLC-coupled bradykinin (BK_2_) receptors [45] contributing together to hypersensitivity to heat [46]. Hence, this evidence supports a possible pharmacogenetic importance. Further evidence about functional associations of *TRPV1* gene variants has been raised in Spanish Caucasian migraine patients in whom the presence of the *TRPV1* rs222741 variant conferred a disease risk [47].

The addition of leukotriene B4 (LTB4) receptors to the *TRPV1* gene panel anticipates a possible pharmacogenetic role in TRPV1 targeting analgesics resulting from recent evidence about a co-expression of the receptors at nociceptive neurons and functional their interplay [8]. LTB4 is a potent proinflammatory agent and its signaling pathway involves two distinct G protein coupled receptors of which BLT1 is a high-affinity and BLT2 a low-affinity LTB4 receptor [48]. The interaction of LTB4 at these receptors is a contributing factor in the pathogenesis of inflammatory diseases [49]. Studies involving the targeted deletion of murine BLT1 and the effect of antagonizing LTB4 receptors in inflammatory models have highlighted the therapeutic potential of BLT receptors with regard to inflammatory diseases [49]. LTB4 has also been shown to activate the TRPV1 channel [50, 51] which leads to excitation of nociceptors and evokes pain-related behaviors [25]. While variants in the two LTB4 receptors potentially affect TRPV1 modulation based analgesic therapies, evidence about functional polymorphisms in these genes is sparse. Studies have suggested a role of polymorphisms overreaching leukotriene pathway genes in determining leukotriene production and susceptibility to allergic disorders, such as inflammatory cell chemotaxis and asthma [52]. Both receptor genes were shown to be polymorphic, in addition, *LTB4R* and *LTB4R2* show splice variations at multiple regions, however, the functional significance has yet to be determined [53].

The present NGS method is suitable for large-scale sequencing of an extended set of human genes involving the main target, *TRPV1*, and recently identified co-players, *LTB4R* and *LTB4R2*. By covering almost, the complete relevant coding and regulatory parts of these genes, the method includes all variants studied so far for functional associations and adds a large amount of genetic information as a basis for complete analysis of human TRPV1 ion channel genetics and its functional consequences. The assay aimed at the complete coding and regulatory information of the selected genes, which regards the increasing acknowledgment of the insufficiency of addressing a limited selection of published functional genetic variants in providing a satisfactory genetic diagnosis of the clinical phenotype. Research interest in the complete genomic information dates back to the seventies of last century when the selective incorporation of chain-terminating dideoxynucleotides by DNA polymerase during *in vitro* DNA replication had been introduced [17, 18]. Techniques significantly improved during the last decades with the development of contemporary machines in the late 1990s re-leased to the market around the year 2005. The term “next generation” DNA sequencing refers to high-throughput technologies capable of parallel analyzes of large numbers of different DNA sequences in a single reaction [54]. NGS has been attributed the potential to accelerate biomedical research [12, 55, 56].

Currently, two commercial NGS platforms are widely used for diagnostic purposes: the MiSeq/HiSeq/NextSeq (Illumina, Hayward, CA, USA) and the Ion Torrent PGM (Life Technologies, Carlsbad, CA, USA). Both platforms combine conceptually similar workflows, starting with the creation of the genetic sample, which commences library preparation involving fragmentation of genomic DNA, purifying to uniform and desired fragment size and ligation to sequencing adapters specific to the platform. Differences apply to the reaction biochemistry and the way how the sequencing information is read [54]. In the present ion semiconductor sequencing method, libraries are immobilized to beads and amplified in microdroplets of aqueous solution and oil using emulsion PCR. Individual nucleotide bases are incorporated via DNA polymerase, which in the case of success triggers the release of a proton. The semiconductor chip that acts as a pH meter [57] providing the final readout. Alternative techniques use the detection of light instead, i.e., from optical fluorescence signals in the case of successful nucleotide incorporation the DNA nucleotide sequence is assembled. The different techniques differ with respect to the obtained throughput and accuracy, but multiple studies have shown that both NGS platforms provide reliable sequencing results in routine clinical diagnostics [58–61] and a recent study came up with a 100% concordance between NGS and an alternative diagnostic approach in mutant allele detection [62].

The high throughput and comprehensive information about DNA sequences are presently reflected in the assay costs. The sequencing of the *TRPV1*, *LTB4R* and *LTB4R2* receptor genes of 80 patients required € 1,500 for the AmpliSeq^™^ custom panel, € 5,880 for library preparation, € 980 for template preparation and € 1,400 for sequencing. In addition, approximately € 600 were spent for consumables and laboratory supplies. With 40 barcoded samples loaded on two chips, respectively, analysis costs for a single patient’s gene sequence were approximately € 130. NGS costs are expected to quickly fall in near future [63]. However, despite this rapid technological progress, the analysis of the generated large data sets remains challenging [64]. As the sequencing process is only the beginning of the procedure, the analysis of the resulting “big data” requires substantial computational power, bioinformatics expertise and “up to date” databases of genomic variations. NGS technologies seem to shift the workload essentially away from the laboratory sample preparation toward various data analysis processes.

We report a NGS assay based on AmpliSeq^™^ libraries and Ion Personal Genome Machine (PGM) suitable for large-scale sequencing of *TRPV1* and functionally related genes. While the aim of assay development had the pharmacogenetics of TRPV1-targeting novel analgesics in mind, the roles of TRPV1 and the two LTB4 receptors are not restricted to this setting. By contrast, the expression of TRPV1 is also observed in non-neuronal sites such as the epithelium of bladder and lungs and in hair cells of the cochlea. At these sites, TRPV1 serves as a potential drug target for treating various diseases such as cystitis, asthma and hearing loss [65].

## Supporting information

S1 Table A list of PCR primer used for the NGS assay. (DOCX)

S2 Table A list of missed parts from the gene panel. (DOCX)

S3 Table The accession numbers of the original data at the BioProject database. (DOCX)
