# Supplementary material for: Next-generation sequencing of the human TRPV1 gene and the regulating co-players LTB4R and LTB4R2 based on a custom AmpliSeq™ panel
Source: PLoS One. 2017 Jun 28;12(6):e0180116. doi: 10.1371/journal.pone.0180116 (PMC5489211; doi:10.1371/journal.pone.0180116)
Supplement: S1 Table — (DOCX) [file pone.0180116.s001.docx]

S1 Table: Ampliseq primer sequences used for the amplification of DNA segments of the human *TRPV1*, *LTB4R LTB4R2* receptor genes suitable for NGS analysis by means of ion semiconductor sequencing.

| Gene | Primer forward start | Primer reverse | Amplicon start | Amplicon stop |
| --- | --- | --- | --- | --- |
| *TRPV1* | TTGACCGCAGGGAGAAGCTCA | CCACCACATCTCCATCCTGATG | 3474824 | 3475046 |
|  | TCTTCATCCTTGCTGGATCCTCT | GCAGCAGCAAGTTGGAGTTTTG | 3495638 | 3495801 |
|  | TCGAGCACTTGCCTCTCTTAAAA | GGGAGGCCAAGAAGTGAATGTTT | 3470220 | 3470344 |
|  | TCCTTGCCATCAGGTGTGTAC | GCCAGGCTACAGAAACAAAGCTAG | 3475437 | 3475705 |
|  | CAGGGAGTAAGGATCCCAGAGAA | TTCCTGCAGAAGAGCAAGAAGCA | 3494152 | 3494526 |
|  | TCACGTATGGTTGCTATGCTTAGG | CAGGTCTTGGCCTATATTCTCCAG | 3491293 | 3491664 |
|  | GGAGTTCTGCAGCAGGAACTT | CCATCGAGAGACGCAACATG | 3493329 | 3493665 |
|  | CCCGAGTATGTATTCAGTGCCTT | ACCCTTGCTGTTCTGCTCTTTT | 3483600 | 3483966 |
|  | GCAGGAGGATGTAGGTGAGAATTACA | CTTGGGATATTGGCTTCTATCCAGTG | 3477004 | 3477302 |
|  | TCAGGGCCAGATTGCTTGATTC | TGTACTTCAGCCACCTCAAGGA | 3480734 | 3481019 |
|  | CGGCCAAGAATGGGCTTTAGTA | CCATTTGAGTTGGCATCCCTGTA | 3512475 | 3512840 |
|  | CCCTGGCTTTGTGACATTTAGC | CTGGATGGAGACCCTAACTCCA | 3495259 | 3495572 |
|  | GGCCACTCACTATGCCATCTTA | CTTTGGGATCCTCTGGGAATCAG | 3500145 | 3500468 |
|  | GCTTTGCAAACTATTTGGTCTCTGT | GAACCACATCCCAAAGGTACCT | 3468509 | 3468881 |
|  | TCCTGGGCTTCATTTACTTTTGCT | ACCTGGCTGTTTTCTTTGATTTTATTCTTT | 3469137 | 3469510 |
|  | AAAAGAATAAAATCAAAGAAAACAAGGGCCT | GGGAAGCGTTCTTGGAAGCA | 3469793 | 3469959 |
|  | CCCATGTCTTGTTGCTGATACTCA | GACGAGCATGTACAATGAGATTCTGAT | 3492860 | 3493227 |
|  | CCACAGGACCTCAAGGTTAAAGG | ACAAGTGGGACAGATTCGTCAAG | 3488812 | 3489171 |
|  | GGGCTAGGCATAAGACCTGAAC | GGTGCAGAGTGTGCAGTATAGA | 3498769 | 3499128 |
|  | CTCCAGCACCGAGTTCTTCTC | CTGGTTGTTGAGGTACTGTCTGA | 3491512 | 3491733 |
|  | CCCAAGTAGGGCTATGATGTGT | CCTAATAACACACTGTCTTTGTGTGTC | 3486516 | 3486868 |
|  | CCCTGTCTCAGGGTCTGAAAGA | GATTTAGTCGTGTTCCCTTGACAAG | 3494397 | 3494736 |
|  | GCCAAGTCCTGGAGCTCATTTC | CTGAGCTGAGAACCAGCAAAGCA | 3480253 | 3480619 |
|  | GGCAGTAGACCAGGAAGTTGAAG | CACTTAAGAGCTTGTCCAGTTTCAC | 3489115 | 3489485 |
|  | CGCCCTTTGGTTTTCTTAAAGAAGT | CTTCCAACCCGTTATTTCCTGTTC | 3493563 | 3493912 |
|  | CAGCATGAGTGAGGTGCTCAA | CGTGGGCATCATCAACGAAGA | 3474631 | 3474897 |
|  | GCTTTCTGCTCCCTCCTCAAATC | TTCAAGGCTGTCTTCATCATCCTG | 3476733 | 3477064 |
|  | CAAGGCCAGGGAGAATACCATG | TATACATAAAGAGTGAGATTTTGCACAGCT | 3480964 | 3481288 |
|  | CTGGCCTCTGGATGGTGATAAC | GCAGTCCTTACTTTGGGAGTCAAC | 3495383 | 3495723 |
|  | GCAGGGTGAGGTTGATAACCAC | ACAAAATCCAAAATCTCGTATAAGCTCAGT | 3496006 | 3496315 |
|  | CCATGAACCTTTCGGCAATGTT | TGCCTAAATCACGGTAGAAGTTCC | 3498975 | 3499339 |
|  | GCATCACTACCAGCATCACTACT | CAGGAAAGCTGTCCACAGTAGTC | 3511658 | 3512007 |
|  | GGGTTCCTAGAAATGGAAGATCTTTCAA | CGCTCGGCCTTCTTTGATTTTA | 3468821 | 3469192 |
|  | AAAAAGAATAAAATCAAAGAAAACAGCCAGG | TGGATGGTCAGTCTCTACTGGG | 3469479 | 3469852 |
|  | GGACAGTGACGGTTGGATGTAC | GCAGACACTGGAAGAACTTTGC | 3469907 | 3470275 |
|  | CCCTTCTGGACACCTCGAATTT | GAGCTTCCTTAAGTGCATGAGGAA | 3475175 | 3475514 |
|  | CGTCATTCCCTTCTTGTTGGTGA | TTGGGCAGAGACAGAGGGAGTTT | 3493137 | 3493491 |
| *LTB4R* | TCAGGAAACCCTTGGTCCTCTA | CTCCGCAGACATAGTGACACAG | 24784789 | 24785152 |
|  | CTCGCGTACCGCACAGTA | CGAGTGCGATGAGCACGTT | 24785314 | 24785677 |
|  | CCCTCTCAAGTTAAACGAACTGAAC | CACTCACAAGGTTGACCAAATTTCAG | 24785889 | 24786163 |
|  | GGCTTATCACTCCAGGTTCTGT | GTTCTCTAAAACAACAGTCATCTCTAGGA | 24786397 | 24786760 |
|  | CCAAAGTATGAGCCAGTGAGAATGAG | ACTCAAAACAAAAATTTGCCCTTTCCT | 24786941 | 24787298 |
|  | CACGGGATTCTTTCTGTCCTCAT | CCTTTCAACTCTCACTTCTCAGGAA | 24782454 | 24782739 |
|  | GGTCAGATTGAAGGAAGGACTTTTTAGT | CCTTGAAGTCTCCTGCAAAAGC | 24782757 | 24783131 |
|  | CGGCCTCAACTTTGTGTGTCTA | CTCCATTCTCTTTGGTAGAAGCCAA | 24783270 | 24783491 |
|  | ATATCAGCATTGTAGCCTCCAATCTG | AGGTCTAGGTGCTGTTTGCTAAATC | 24783747 | 24784119 |
|  | ACTGCTCCCTTTTTCCTTCACTTC | GCACAGGCTCATGTTCGTTTTC | 24785065 | 24785361 |
|  | CGTGGTGAACCTGGCTGAG | AGGAAAGTGCGCCTCCTTC | 24785571 | 24785943 |
|  | ACCACAACCTGCCAGGAATTTTTA | TCTTTCCTATCTTCTCACAGCCTACTATT | 24783079 | 24783331 |
|  | TGTCCTCTGCTCTGTGGTACTT | CTTTGCACCTTCTTTCATGGCT | 24784062 | 24784298 |
|  | CCCACAGGCAGCTTTAACCATT | ACAGCAGTGCTGTTTTCTCAGAA | 24786105 | 24786452 |
|  | CAAAGATTCCCAAAAGTGAGAGGGAT | ACAGATGCTGGTATATGGGTCTTGA | 24786695 | 24787002 |
|  | GACCTCAGTGGCCACCATTAT | GGAGCAGATCACTTGATGTTAGCAG | 24782683 | 24783039 |
|  | GCATTCTGTGTGATACCAAGGAGA | AGGAGGTATGGTAGCCAGAGAAG | 24783432 | 24783806 |
|  | CTCTACTTTAGCGACTGCTCACA | GTGCAACAAAGGAAGTTAAGGAACCT | 24782161 | 24782513 |
| *LTB4R2* | GAAGGATGTCGGTCTGCTACC | CGTAGTACACCGCCTTGCA | 24779866 | 24780168 |
|  | CCTGAGCCTGGAGACTCTGA | GCGGTGAAGACGTAGAGCA | 24780413 | 24780745 |
|  | GAGGTCAGTGTTCTGGGACATT | CTAGGTGGCTTCAGTACAACTCAG | 24781008 | 24781309 |
|  | CCACTTCAGCTTGTGCTGTTTC | TCACTATCTCTACCACCACTCCTCTA | 24779208 | 24779580 |
|  | GCTCTTTGTGGCCTTCCTGAC | TCCACAGGTGGCGGTAGAC | 24780098 | 24780360 |
|  | CCAGACTAGAGGAGTGGTGGTA | CAGCTCAGCAGTGTCTCGTT | 24779549 | 24779917 |
|  | GCCTTGGCCTTCTTCAGTTCTAG | AGCCAGATCCAAACTCTAGTCAAAG | 24780693 | 24781065 |
