# Supplementary material for: Next-generation sequencing of the human TRPV1 gene and the regulating co-players LTB4R and LTB4R2 based on a custom AmpliSeq™ panel
Source: PLoS One. 2017 Jun 28;12(6):e0180116. doi: 10.1371/journal.pone.0180116 (PMC5489211; doi:10.1371/journal.pone.0180116)
Supplement: S2 Table — (DOCX) [file pone.0180116.s002.docx]

S2 Table: SNPs listed at the dbSNP database at <https://www.ncbi.nlm.nih.gov/SNP/> that are missing from the present *TRPV1*, *LTB4R* and *LTB4R2* NGS gene panel.

| Chromosome | Chr start | Chr end | Gene | dbSNP | Molecular consequence |
| --- | --- | --- | --- | --- | --- |
| Chr14 | 24780345 | 24780346 | *LTB4R2* | rs771073522 | missense |
| Chr14 | 24780346 | 24780347 | *LTB4R2* | rs774566419 | coding-synon |
| Chr14 | 24780347 | 24780348 | *LTB4R2* | rs759602378 | missense |
| Chr14 | 24780349 | 24780350 | *LTB4R2* | rs760533968 | coding-synon |
| Chr14 | 24780355 | 24780356 | *LTB4R2* | rs767626246 | coding-synon |
| Chr14 | 24780358 | 24780359 | *LTB4R2* | rs776762055 | near-gene-5 |
| Chr14 | 24780363 | 24780364 | *LTB4R2* | rs1950504 | near-gene-5 |
| Chr14 | 24780365 | 24780366 | *LTB4R2* | rs76678850 | missense |
| Chr14 | 24780368 | 24780369 | *LTB4R2* | rs368558186 | near-gene-5 |
| Chr14 | 24780376 | 24780377 | *LTB4R2* | rs758151831 | missense |
| Chr14 | 24780382 | 24780383 | *LTB4R2* | rs765948741 | near-gene-5 |
| Chr14 | 24780387 | 24780388 | *LTB4R2* | rs371024231 | near-gene-5 |
| Chr14 | 24780388 | 24780389 | *LTB4R2* | rs754503961 | coding-synon |
| Chr14 | 24780390 | 24780406 | *LTB4R2* | rs759432777 | near-gene-5 |
| Chr14 | 24780393 | 24780394 | *LTB4R2* | rs146489502 | near-gene-5 |
| Chr14 | 24780397 | 24780398 | *LTB4R2* | rs35322717 | coding-synon |
| Chr14 | 24780399 | 24780400 | *LTB4R2* | rs756642549 | missense |
| Chr14 | 24780403 | 24780404 | *LTB4R2* | rs372157907 | coding-synon |
| Chr14 | 24780406 | 24780407 | *LTB4R2* | rs560930276 | coding-synon |
| Chr14 | 24780409 | 24780410 | *LTB4R2* | rs779062016 | coding-synon |
| Chr14 | 24780412 | 24780413 | *LTB4R2* | rs746084677 | coding-synon |
| Chr14 | 24780414 | 24780415 | *LTB4R2* | rs772087412 | missense |
| Chr14 | 24780419 | 24780420 | *LTB4R2* | rs775746953 | missense |
| Chr14 | 24780424 | 24780425 | *LTB4R2* | rs760791457 | missense |
| Chr14 | 24780426 | 24780428 | *LTB4R2* | rs374257326 | near-gene-5 |
| Chr14 | 24780427 | 24780428 | *LTB4R2* | rs769864927 | coding-synon |
| Chr17 | 3469495 | 3469496 | *TRPV1* | rs577837801 | untranslated-3 |
| Chr17 | 3495828 | 3495829 | *TRPV1* | rs796596375 | intron |
| Chr17 | 3495831 | 3495832 | *TRPV1* | rs748019950 | intron |
| Chr17 | 3495854 | 3495855 | *TRPV1* | rs556690488 | intron |
| Chr17 | 3495882 | 3495883 | *TRPV1* | rs771110957 | intron |
| Chr17 | 3495900 | 3495901 | *TRPV1* | rs774542792 | intron |
| Chr17 | 3495910 | 3495911 | *TRPV1* | rs573864007 | intron |
| Chr17 | 3495923 | 3495924 | *TRPV1* | rs542874954 | intron |
| Chr17 | 3495927 | 3495928 | *TRPV1* | rs559108130 | intron |
| Chr17 | 3495934 | 3495935 | *TRPV1* | rs56365804 | intron |
| Chr17 | 3495953 | 3495954 | *TRPV1* | rs180860445 | intron |
| Chr17 | 3495954 | 3495955 | *TRPV1* | rs373288915 | intron |
| Chr17 | 3495966 | 3495967 | *TRPV1* | rs545161291 | intron |
