# Supplementary material for: Next-generation sequencing of the human TRPV1 gene and the regulating co-players LTB4R and LTB4R2 based on a custom AmpliSeq™ panel
Source: PLoS One. 2017 Jun 28;12(6):e0180116. doi: 10.1371/journal.pone.0180116 (PMC5489211; doi:10.1371/journal.pone.0180116)
Supplement: S3 Table — (DOCX) [file pone.0180116.s003.docx]

S3 Table: Accession numbers for our sequencing data for the BioProjekt “PRJNA387106”. Data can be accessed at the BioProject database at <https://www.ncbi.nlm.nih.gov/Traces/study/?acc=SRP107294>.

| BioSample | Run | Experiment | Sample name | MBases | MBytes |
| --- | --- | --- | --- | --- | --- |
| SAMN07139129 | SRR5574546 | SRX2833015 | D005_TRP_Pat75 | 7 | 4 |
| SAMN07139097 | SRR5574547 | SRX2833014 | D005_TRP_Pat43 | 6 | 3 |
| SAMN07139114 | SRR5574551 | SRX2833010 | D005_TRP_Pat60 | 47 | 25 |
| SAMN07139109 | SRR5574550 | SRX2833011 | D005_TRP_Pat55 | 30 | 15 |
| SAMN07139134 | SRR5574549 | SRX2833012 | D005_TRP_Pat80 | 2 | 1 |
| SAMN07139126 | SRR5574548 | SRX2833013 | D005_TRP_Pat72 | 10 | 6 |
| SAMN07139108 | SRR5574559 | SRX2833002 | D005_TRP_Pat54 | 26 | 14 |
| SAMN07139094 | SRR5574558 | SRX2833003 | D005_TRP_Pat40 | 2 | 1 |
| SAMN07139106 | SRR5574557 | SRX2833004 | D005_TRP_Pat52 | 9 | 5 |
| SAMN07139105 | SRR5574556 | SRX2833005 | D005_TRP_Pat51 | 13 | 7 |
| SAMN07139112 | SRR5574555 | SRX2833006 | D005_TRP_Pat58 | 40 | 21 |
| SAMN07139111 | SRR5574554 | SRX2833007 | D005_TRP_Pat57 | 7 | 4 |
| SAMN07139110 | SRR5574553 | SRX2833008 | D005_TRP_Pat56 | 2 | 1 |
| SAMN07139093 | SRR5574552 | SRX2833009 | D005_TRP_Pat39 | 3 | 2 |
| SAMN07139072 | SRR5574575 | SRX2832986 | D005_TRP_Pat18 | 35 | 18 |
| SAMN07139071 | SRR5574574 | SRX2832987 | D005_TRP_Pat17 | 23 | 12 |
| SAMN07139070 | SRR5574573 | SRX2832988 | D005_TRP_Pat16 | 1 | 1 |
| SAMN07139069 | SRR5574572 | SRX2832989 | D005_TRP_Pat15 | 1 | 1 |
| SAMN07139068 | SRR5574571 | SRX2832990 | D005_TRP_Pat14 | 4 | 2 |
| SAMN07139067 | SRR5574570 | SRX2832991 | D005_TRP_Pat13 | 12 | 6 |
| SAMN07139066 | SRR5574569 | SRX2832992 | D005_TRP_Pat12 | 5 | 3 |
| SAMN07139065 | SRR5574568 | SRX2832993 | D005_TRP_Pat11 | 20 | 10 |
| SAMN07139086 | SRR5574567 | SRX2832994 | D005_TRP_Pat32 | 1 | 0 |
| SAMN07139085 | SRR5574566 | SRX2832995 | D005_TRP_Pat31 | 1 | 0 |
| SAMN07139088 | SRR5574565 | SRX2832996 | D005_TRP_Pat34 | 9 | 5 |
| SAMN07139087 | SRR5574564 | SRX2832997 | D005_TRP_Pat33 | 5 | 3 |
| SAMN07139090 | SRR5574563 | SRX2832998 | D005_TRP_Pat36 | 0 | 0 |
| SAMN07139089 | SRR5574562 | SRX2832999 | D005_TRP_Pat35 | 4 | 2 |
| SAMN07139074 | SRR5574561 | SRX2833000 | D005_TRP_Pat20 | 2 | 1 |
| SAMN07139073 | SRR5574560 | SRX2833001 | D005_TRP_Pat19 | 8 | 4 |
| SAMN07139121 | SRR5574604 | SRX2832957 | D005_TRP_Pat67 | 1 | 0 |
| SAMN07139122 | SRR5574603 | SRX2832958 | D005_TRP_Pat68 | 0 | 0 |
| SAMN07139119 | SRR5574602 | SRX2832959 | D005_TRP_Pat65 | 0 | 0 |
| SAMN07139102 | SRR5574601 | SRX2832960 | D005_TRP_Pat48 | 1 | 1 |
| SAMN07139117 | SRR5574600 | SRX2832961 | D005_TRP_Pat63 | 1 | 1 |
| SAMN07139118 | SRR5574599 | SRX2832962 | D005_TRP_Pat64 | 1 | 1 |
| SAMN07139115 | SRR5574598 | SRX2832963 | D005_TRP_Pat61 | 21 | 11 |
| SAMN07139116 | SRR5574597 | SRX2832964 | D005_TRP_Pat62 | 21 | 11 |
| SAMN07139095 | SRR5574596 | SRX2832965 | D005_TRP_Pat41 | 21 | 11 |
| SAMN07139123 | SRR5574595 | SRX2832966 | D005_TRP_Pat69 | 2 | 1 |
| SAMN07139096 | SRR5574594 | SRX2832967 | D005_TRP_Pat42 | 48 | 25 |
| SAMN07139128 | SRR5574593 | SRX2832968 | D005_TRP_Pat74 | 26 | 14 |
| SAMN07139092 | SRR5574592 | SRX2832969 | D005_TRP_Pat38 | 1 | 0 |
| SAMN07139107 | SRR5574591 | SRX2832970 | D005_TRP_Pat53 | 2 | 1 |
| SAMN07139091 | SRR5574590 | SRX2832971 | D005_TRP_Pat37 | 0 | 0 |
| SAMN07139058 | SRR5574589 | SRX2832972 | D005_TRP_Pat4 | 27 | 14 |
| SAMN07139057 | SRR5574588 | SRX2832973 | D005_TRP_Pat3 | 15 | 8 |
| SAMN07139056 | SRR5574587 | SRX2832974 | D005_TRP_Pat2 | 14 | 7 |
| SAMN07139055 | SRR5574586 | SRX2832975 | D001_TRP_Pat1 | 4 | 2 |
| SAMN07139062 | SRR5574585 | SRX2832976 | D005_TRP_Pat8 | 6 | 3 |
| SAMN07139061 | SRR5574584 | SRX2832977 | D005_TRP_Pat7 | 2 | 1 |
| SAMN07139060 | SRR5574583 | SRX2832978 | D005_TRP_Pat6 | 31 | 16 |
| SAMN07139059 | SRR5574582 | SRX2832979 | D005_TRP_Pat5 | 28 | 14 |
| SAMN07139120 | SRR5574581 | SRX2832980 | D005_TRP_Pat66 | 10 | 5 |
| SAMN07139124 | SRR5574580 | SRX2832981 | D005_TRP_Pat70 | 10 | 5 |
| SAMN07139064 | SRR5574579 | SRX2832982 | D005_TRP_Pat10 | 0 | 0 |
| SAMN07139130 | SRR5574578 | SRX2832983 | D005_TRP_Pat76 | 5 | 3 |
| SAMN07139132 | SRR5574577 | SRX2832984 | D005_TRP_Pat78 | 15 | 8 |
| SAMN07139131 | SRR5574576 | SRX2832985 | D005_TRP_Pat77 | 6 | 3 |
| SAMN07139063 | SRR5574545 | SRX2833016 | D005_TRP_Pat9 | 1 | 1 |
| SAMN07139127 | SRR5574544 | SRX2833017 | D005_TRP_Pat73 | 23 | 12 |
| SAMN07139101 | SRR5574543 | SRX2833018 | D005_TRP_Pat47 | 0 | 0 |
| SAMN07139113 | SRR5574622 | SRX2832939 | D005_TRP_Pat59 | 40 | 21 |
| SAMN07139125 | SRR5574621 | SRX2832940 | D005_TRP_Pat71 | 9 | 5 |
| SAMN07139098 | SRR5574620 | SRX2832941 | D005_TRP_Pat44 | 0 | 0 |
| SAMN07139133 | SRR5574619 | SRX2832942 | D005_TRP_Pat79 | 16 | 8 |
| SAMN07139099 | SRR5574618 | SRX2832943 | D005_TRP_Pat45 | 9 | 5 |
| SAMN07139083 | SRR5574617 | SRX2832944 | D005_TRP_Pat29 | 0 | 0 |
| SAMN07139084 | SRR5574616 | SRX2832945 | D005_TRP_Pat30 | 1 | 1 |
| SAMN07139103 | SRR5574615 | SRX2832946 | D005_TRP_Pat49 | 5 | 3 |
| SAMN07139104 | SRR5574614 | SRX2832947 | D005_TRP_Pat50 | 14 | 8 |
| SAMN07139100 | SRR5574613 | SRX2832948 | D005_TRP_Pat46 | 0 | 0 |
| SAMN07139077 | SRR5574612 | SRX2832949 | D005_TRP_Pat23 | 0 | 0 |
| SAMN07139078 | SRR5574611 | SRX2832950 | D005_TRP_Pat24 | 1 | 1 |
| SAMN07139075 | SRR5574610 | SRX2832951 | D005_TRP_Pat21 | 21 | 11 |
| SAMN07139076 | SRR5574609 | SRX2832952 | D005_TRP_Pat22 | 0 | 0 |
| SAMN07139081 | SRR5574608 | SRX2832953 | D005_TRP_Pat27 | 13 | 7 |
| SAMN07139082 | SRR5574607 | SRX2832954 | D005_TRP_Pat28 | 30 | 15 |
| SAMN07139079 | SRR5574606 | SRX2832955 | D005_TRP_Pat25 | 26 | 13 |
| SAMN07139080 | SRR5574605 | SRX2832956 | D005_TRP_Pat26 | 16 | 8 |
